# Investigation of Balance Function Using Dynamic Posturography under Electrical-Acoustic Stimulation in Cochlear Implant Recipients

**DOI:** 10.1155/2010/978594

**Published:** 2010-06-28

**Authors:** B. Schwab, M. Durisin, G. Kontorinis

**Affiliations:** Department of Otolaryngology, Medical University of Hannover, 30625 Hannover, Germany

## Abstract

*Introduction*. The purpose of the present study is to investigate the effect of electrical-acoustic stimulation on vestibular function in CI patients by using the EquiTest and to help answer the question of whether electrically stimulating the inner ear using a cochlear implant influences the balance system in any way. *Material and Methods*. A test population (*n* = 50) was selected at random from among the cochlear implant recipients. Dynamic posturography (using the EquiTest) was performed with the device switched off an switched on. *Results*. In summary, it can be said that an activated cochlear implant affects the function of the vestibular system and may, to an extent, even lead to a stabilization of balance function under the static conditions of dynamic posturography, but nevertheless also to a significant destabilization. Significant improvements in vestibular function were seen mainly in equilibrium scores under conditions 4 and 5, the composite equilibrium score, and the vestibular components as revealed by sensory analysis. *Conclusions*. Only under the static conditions are significantly poorer scores achieved when stimulation is applied. It may be that the explanation for any symptoms of dizziness lies precisely in the fact that they occur in supposedly noncritical situations, since, when the cochlear implant makes increased demands on the balance system, induced disturbances can be centrally suppressed.

## 1. Introduction

Cochlear implant recipients often complain of postoperative symptoms of dizziness [1–4]. Although the auditory and vestibular systems are clearly distinct from one another, the mechanisms of neural transmission are identical. For this reason the electrical stimulation through the agency of the cochlear implant may have an effect both on the auditory and vestibular systems. 

Several studies on the effect of electrical stimulation by the cochlear implant were carried out. Ito in 1998 described in his study that 18% of 55 cochlear implant recipients saw connection between dizziness and the activation of the CI. This gave grounds for supposing that the electricity spreads diffusely and could therefore stimulate the nerve endings of the vestibular nerve [5]. 

Bance et al.tested 17 patients for spontaneous nystagmus using video nystagmography. Only one patient produced eye movements under electrical stimulation by the cochlear implant, although no discomfort was reported [6]. In this study, the proportion of cases in which the cochlear implant had a detrimental effect on the vestibular organ was 6%, which closely corresponds to the findings of the investigation by Shea into the stimulation of the facial nerve by the cochlear implant.

 In order to verify this possible effect on a larger patient population, we used the EquiTest to assess 50 postlingually deafened patients under acoustic stimulation markedly above the threshold, and compared the results with those in the stimulation-free situation.

The purpose of the present study is to investigate the possible effect of electrical-acoustic stimulation on vestibular function in CI patients by using the EquiTest. For this aim a large test population was used, allowing statistical analysis to be made. 

## 2. Material and Methods

### 2.1. Patients

A test population (*n* = 50) was selected at random from among the cochlear implant recipients implanted at the Medical University of Hannover's Department of Otolaryngology. The test population comprised 27 female and 23 male postlingually deafened adult patients with an average age of 47 years. The average age at onset of deafness in 28 patients with acute hearing deterioration was 39.5 years. In 22 patients the time of deafness could not be precisely pinpointed, as the clinical course had been characterized by continuous progression. The average age at implantation was 46.3 years. The cause of deafness was unknown in most cases (*n* = 26). Further causes were sudden hearing loss (*n* = 7), meningitis and otosclerosis (both *n* = 3), asphyxia and hereditary causes (both *n* = 2), destructive choleastoma, mumps, diphtheria, fibroinflammatory pseudotumors, chronic otitis media, hypoglycemic coma, and rhesus incompatibility (all *n* = 1). None of the patients show any physical and mental disabilities or inadequate compliance which could negatively affect results of testing using dynamic posturography. 

The majority of the patients (*n* = 43) were tested between the sixth and eighth weeks following implantation. Six of the patients had been using a cochlear implant for a fairly long period of time (on average 4.75 years). Detailed patient's medical history was taken in relation to any symptoms of dizziness experienced before and after the surgery.

Three models of CI were represented: 42 patients were Clarion (Advanced Bionics Corporation, Sylmar, California, USA) recipients, four had the Nucleus 22 and four the Nucleus 24 (Cochlear Limited, Lane Cove, Australia).

All patients were tested preoperatively by using nystagmus test, Roberg test, Unterberger stepping test, and caloric test. Postoperatively was the EquiTest performed.

### 2.2. Methods

#### 2.2.1. Nystagmus Test, Roberg Test, Unterberger Stepping Test, and Caloric Test

A nystagmus test, the Romberg test, the Unterberger stepping test and caloric testing were carried out as part of the routine preoperative procedure; reference was therefore made to the results of these tests as the basis for assessing preoperative vestibular function. 

#### 2.2.2. The EquiTest

The EquiTest assesses both the balance system as a whole and its individual components—that is, the vestibular, visual and somatosensory systems—in their own right. The EquiTest protocol was developed by Nashner et al. [7–9] and has been in commercial use since 1986. It comprises the Sensory Organisation Test (SOT) and the Motor Control Test (MCT). 

**Figure 1:**
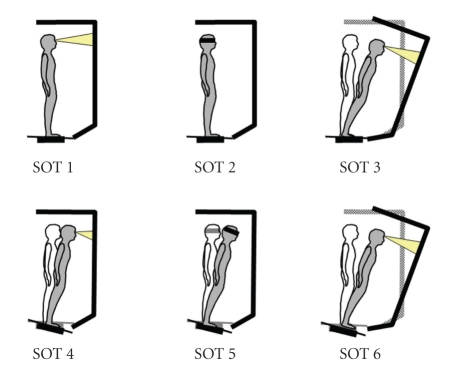
Test conditions SOT 1 to 6 under which equilibrium score is determined: under condition 1 (eyes open), and condition 2 (eyes closed), both the platform and the surround remain immobilized. Under condition 3, the surround moves. Under condition 4, the platform moves and the surround remains fixed. Under condition 5, the platform moves while the subject keeps his/her eyes closed. Under condition 6, both the surround and the platform move.

The SOT involves six test conditions of increasing difficulty. In condition 1 (SOT 1) is patient in the starting position with open eyes, in condition 2 (SOT 2) with closed eyes. For SOT 1 and 2 both the platform and the surround remain immobilized. In condition 3 (SOT 3) is patient in starting position, however the surround moves, in condition 4 (SOT 4) the platform moves, however the surround remains fixed. In condition 5 (SOT 5) the paltform moves while the subject keeps his/her eyes closed, and in condition 6 (SOT 6) both the surround and the platform move (Figure 1). Adaption scores of 6 conditions as well as the composite equilibrium were evaluated. The composite equilibrium score is a mathematical-analytic indicator of balance. It is calculated by independently averaging the scores achieved under conditions 1 and 2, adding these two values to the sum of all three scores under sensory conditions 3, 4, 5, and 6, and then dividing this sum by the total number of trials. The highest possible score is 100. It is the best means of providing an overall impression of how an individual organizes sensory information. 

In sensory analysis, use of an algorithm enables the balance functions (visual, vestibular and somatosensory) to be considered separately (Table 1). The term “visual preference” is also introduced in this connection; this describes the ability to suppress visual information perceived as incorrect.

The MCT assesses automatic motor responses. A sequence of unexpected forward and backward translational movements and slight tilting movements of the platform (also forward and backward) elicits automatic, evaluate muscular postural responses. Measurements enable to evaluate the symmetry of weight distribution, the response speed (i.e., latency) and the intensity and symmetry. The main purpose of weight symmetry testing, which detects a shift in the center of gravity in both the static and dynamic situations, is to aid the correct analysis of the latency measurements. If the center of gravity is displaced to one side this may indicate either false adaptation or a musculoskeletal deficiency, such as muscular weakness or an orthopedic problem. If the center of gravity is shifted, this compounds the patient's difficulty in maintaining stability: larger movements are only poorly tolerated. Unilateral latency abnormalities, that is, extended period, indicate a localized disorder in the spinal cord, brainstem or subcortex. Latency is defined as the time (milliseconds) between the onset of a horizontal movement of the support surface and the patient's active response to this movement. If such delays are observed bilaterally, then the disorder is global and central. Greater latencies in only one direction point to a disorder in the efferent limb. A score of 100 indicates that the weight is evenly distributed over both legs. Theoretically possible scores range from 0 to 200, with the extreme value indicating that weight is placed on only one leg.

The strategy analysis was performed in oder to evaluate the relative contributions of movement about the ankles and the hips that are required to maintain balance during the test. The sole use of the ankles to keep balance generates a score of 100%, whereas if only the hips are used the resulting score is 0%. Normally only ankle movements are required to maintain balance. It is not until the limits of the capacity for compensation are reached that hip movements are also employed to achieve compensation.

### 2.3. Procedure for the EquiTest

The patients were familiarized with the test procedure and secured using a safety harness. The EquiTest assessment was first carried out with the cochlear implant switched off. The room was not sound-isolated, but there were no sources of noise. Before each section of the trial, the patients were given written instructions detailing what would follow. After a single test run there was a break of 10–15 minutes' duration before the second test run was carried out with the cochlear implant switched on. With a direct cable connection to the speech processor established, a CD player was used to play white noise as per the CCITT G.227 standard (“Comité Consultativ International Télégraphique et Téléphonique” = Consultative Committee for International Telegraph and Telephone), for which the patients were able to adjust the volume to the subjective setting “very loud, but not unpleasant”.

### 2.4. Data Collection and Statistical Analysis

The values for weight symmetry and latency were taken for the various backward and forward translational movements. The composite values for both equilibrium score and latency were incorporated into the analysis. Means were also determined from the six adaptation scores. Both weight symmetry and adaptation scores that fell outside the normal range were also included. 

The results from the EquiTest under acoustic stimulation were compared with the results without stimulation and transferred to the Excel spreadsheet program, taking into account the requirements of data protection. The statistical analysis of the data was carried out using the Statistical Program for Social Science (SPSS). The Wilcoxon test was used in all cases to determine significance. The significance level was set at 5%  (*P* < .05); *P* < .01 is deemed highly significant.

## 3. Results

### 3.1. Preoperative Status of Vestibular Organ

In 58%  (*n* = 29) of the patients studied, the vestibular organ had a normal level of excitability and no pathological nystagmus was present.

Provocation nystagmus was found in 15 patients (30%). Nystagmus abnormality was classifiable as pronounced (i.e., nystagmus of 6–15 beats/minute was inducible in one of the six defined positions) in six patients and as extreme (nystagmus of more than 30 beats/minute in several positions) in nine. In caloric testing, the response to excitation was either reduced or absent in eight patients (16%). Thus, the peripheral vestibular organ was nonexcitable in three patients, possibly excitable in three, and hypoexcitable in two. In most of these individuals, however, a pathological response was seen in either caloric or nystagmus testing, but not both; only two patients showed both abnormal nystagmus and a pathologically abnormal caloric response. In anamnesis, 36 patients (72%) reported that they had no vestibular symptoms and 14 (28%) affirmed that they did, although most described these as only temporary. Of the latter, nine correlated with a pathologically abnormal nystagmic or caloric response.

### 3.2. Postoperative Status of Vestibular Organ

Postoperatively, vestibular abnormality was observed in only one patient; here the status of the preoperatively normal vestibular organ was classified as “possibly excitable”, but no pathological nystagmus was detectable. Postoperatively 33 patients (66%) said they had no vestibular symptoms, whereas 17 (34%) reported temporary symptoms of dizziness; of these, only eight patients (16%) also showed corresponding symptoms prior to surgery. Three patients (5%) complained of sensations such as parasthesia and irritation of the facial nerve while the cochlear implant was activated.

### 3.3. The Sensory Organisation Test (SOT)

**Figure 2:**
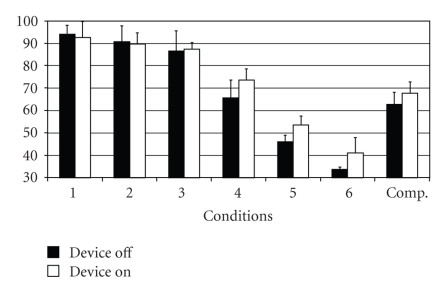
Equilibrium score.

**Figure 3:**
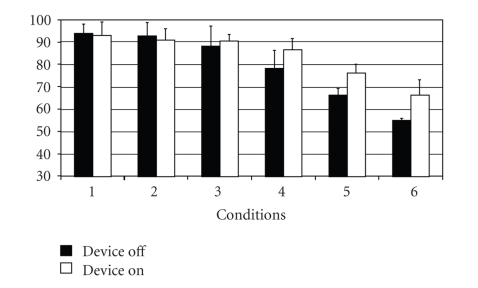
Strategy analysis.

Significantly poorer equilibrium scores were obtained under conditions 1 and 2 (Figure 1) with, accordingly, lower strategy scores under the same two conditions when electrical stimulation was applied (Figure 2). Under condition 3 of the Sensory Organization Test electrical stimulation produced no significant changes compared to the same test without stimulation. There was a remarked improvement in results under electrical stumulation in condition 4 and 5. The strategy analysis results obtained under conditions 4 to 6 were also significantly better. The functions of the somatosensory system and the visual preference, as revealed by sensory analysis, remain approximately the same under electrical stimulation (Figure 3). Poor visual scores are also achieved when, with the support surface in motion, the vestibular system—as opposed to the visual system—assumes a dominant function.

### 3.4. The Motor Control Test (MOT)

**Figure 4:**
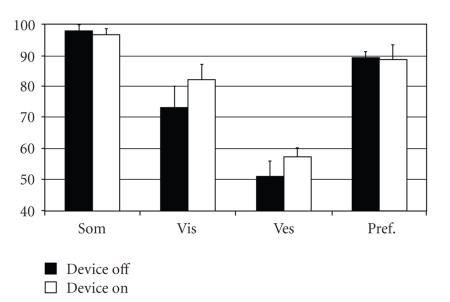
Sensory analysis of the overall test population.

**Figure 5:**
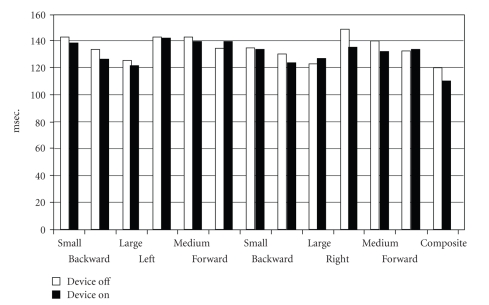
Latency periods exhibited by the overall test population for forward and backward translational movements (separate data for each foot) and for small, medium, and large movements of the force-plate.

Taking the test population as a whole, the latency periods for forward and backward translational movements of the posturography force-plate showed no significant differences (Figure 4). The values for weight symmetry (two-scale test) reveal significant differences in terms of a better average distribution of body weight between the two posturography force-plates (Figure 5) with the CI activated. The dominating dextroposition of the body's center of gravity with the CI switched off is striking, an effect still observed—albeit less markedly—under acoustic-electrical stimulation.

## 4. Discussion

Postoperative balance disorders are widely reported in cochlear implant recipients. Differnt etiologies are postulated for these postoperative symptoms of dizziness. Van den Broek et al. describes in his study perilymphatic fistula induced by cochlear fenestration or a disruption of endolymphatic flow caused by the electrode itself, which could lead to an endolymphatic hydrops, similar to Ménière`s disease [3]. A mechanical irritation of the membranous labyrinth or the labyrinthitis triggered by a foreign body in the cochlea could also caused vestibular disoder in patients with cochlear implant [4]. Thus, hyporeflexia of the vestibular organ may be caused either by intraoperative damage [1, 4, 10–12] or by a disorder that existed preoperatively [13, 14]. 

The effect of the electrical stimulation on the vestibular system could be shown in different studies. As early as the beginning of the 19th century Ritter and Augustin independently published reports of dizziness symptoms that were triggered by electrical stimulation of the moistened outer ear [15, 16]. At higher currents eye movements can be induced, the cause presumably being electrical stimulation of the vestibular structures in the semicircular canal system [17–19]. In experiments on cats (involving stimulation of the round window) a lower threshold was required for stimulating vestibular fibers than auditory nerve fibers [20]. These trials were facilitated by the fact that it is possible in animal experiments to directly stimulate vestibular structures electrically. In guinea pigs vestibular potentials were produced by electrical stimulation of the round window [21]. It is also known that, with the cochlear implant activated, current may also spread beyond the cochlea. This was shown by cochlear implant-induced stimulation of the facial and glossopharyngeal nerves, during which patients complained of pain [22].

In the present study under the dynamic/sensory conditions 4 and 5, in which primarily the vestibular organ itself is tested (both including and excluding the visual system), there was a marked improvement in results. Here it is specifically the somatosensory system that is targeted for irritation by the movement of the platform, this must then be centrally compensated for. 

Visual preference describes the extent to which the patient relies on visual input to maintain balance, even when this information is incorrect. Therefore, even poor scores were obtained, the cause would not be disruption to the individual components; rather, an adaptive problem would be the likely explanation. In this study, however, the visual and vestibular functions improved significantly. The vestibular component of the balance system is not as sensitive as the visual or somatosensory aspects. Nevertheless, the EquiTest does not distinguish between a peripheral and a central component. Moreover, the test is unable to reveal vestibular disorders that are well compensated for. Poor visual scores are also achieved when, with the support surface in motion, the vestibular system—as opposed to the visual system—assumes a dominant function.

In addition, enhanced attentiveness on the part of the patients may have led to an improvement in the dynamic test results. An alternative hypothesis is that acoustic orientation during stimulation by the cochlear implant brings about stabilization in vestibular performance. Significant improvements in vestibular function were seen mainly in equilibrium scores under conditions 4 and 5, the composite equilibrium score, and the vestibular components as revealed by sensory analysis. This effect could be caused by stimulation of the inhibitory parts of the vestibular organ, explainable in terms of their having greater sensitivity than the excitatory portions. 

Only under the static conditions are significantly poorer scores achieved when stimulation is applied: both equilibrium scores and strategy scores under conditions 1 and 2, and the function of the somatosensory system (as revealed by sensory analysis), are detrimentally affected. In principle the first two conditions represent an objectification of conventional vestibulospinal tests such as the Romberg and Unterberger tests. It can therefore be assumed that vestibulospinal functions are influenced. Another conceivable cause is irritation during the initial phase, since conditions 1 and 2 of the SOT are applied at the start of the test sequence. However, none of the patients report substimulatory sensations when switching on the cochlear implant or during the fitting phase. It may be that the explanation for any symptoms of dizziness lies precisely in the fact that they occur in supposedly noncritical situations, since, when the cochlear implant makes increased demands on the balance system, induced disturbances can be centrally suppressed.

This indicates that the suppression of automatic responses to disruptive environmental influences is largely attributable to the somatosensory system. Similar results could be shown in a study byEisenberg,22 patients were tested using ENG (spontaneous nystagmus, postural testing, eye-tracking, thermal testing), coordination tests, and posturography [23]. It was shown that a single-electrode implant did not significantly disrupt the balance system. Indeed, the patients studied actually showed a subjective improvement in postural stability through electrical stimulation, as was corroborated in the present study with a representative number of subjects.

When the results are broken down by preoperative caloric response, strategy analysis reveals a significant improvement under condition 3 in those patients with a normal preoperative caloric response. In the attempt to maintain balance under stimulation, the patients make greater use of ankle movements than hip movements, as is the case in unimpaired individuals. No significant differences became apparent in the preoperative nystagmus test and in the questioning of patients as to their symptoms prior to the operation.

It is clear that dynamic posturography is a technique which allows the effects of electrical stimulation by the cochlear implant on the balance-maintaining system to be demonstrated. In the present study could be shown, that an activated cochlear implant may, to an extent, even lead to a stabilization of balance function under the static conditions of dynamic posturography, but nevertheless also to a significant destabilization. 

## 5. Conclusions

In summary, it can be affirmed that in our study electrical stimulation affect the function of the vestibular system and especially under the challenging test conditions of the EquiTest—actually even led to vestibular stabilization. To an extent, a learning effect may be a contributory factor here, since testing under stimulation was carried out subsequent to testing without stimulation.

This provides at least a partial explanation for the occasionally reported vestibular symptoms experienced by cochlear implant recipients. It also appears that, in many cases, these problems are additionally caused by a preoperative impairment of the vestibular system, attributable to the primary condition that led to deafness.

## Figures and Tables

**Figure 6:**
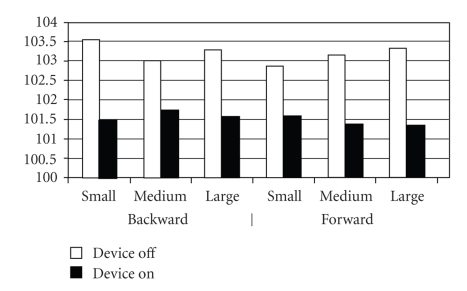
Weight symmetry results for all patients (small, medium, and large movements of the posturography platform).

**Table 1:** Algorithms for calculating the individual components of balance (visual, somatosensory, vestibular, and visual preference) and short explanation.

	Comparison	Short explanation for functional relevance
Somatosensory system (SOM)	Quotient	Patient's ability to use input from the *somatosensory system* to maintain balance
	SOT2/SOT1	
Visual system (VIS)	Quotient	Patient's ability to use input from the *visual system* to maintain balance
	SOT4/SOT1	
Vestibular system (VEST)	Quotient	Patient's ability to use input from the *vestibular system* to maintain balance
	SOT5/SOT1	
Visual preference (PREF)	Quotient	The degree to which a patient relies on visual information to maintain balance, even when the information is incorrect.
	SOT3+SOT6/SOT2+SOT5	
